# Machine learning shows that the Covid-19 pandemic is impacting U.S. public companies unequally by changing risk structures

**DOI:** 10.1371/journal.pone.0269582

**Published:** 2022-06-22

**Authors:** Likun Cao, Jie Ren

**Affiliations:** 1 Division of the Social Science, The University of Chicago, Chicago, IL, United States of America; 2 School of Economics and Management, University of Science and Technology Beijing, Beijing, China; LUMSA: Libera Universita Maria Santissima Assunta, ITALY

## Abstract

Covid-19 has impacted the U.S. economy and business organizations in multiple ways, yet its influence on company fundamentals and risk structures have not been fully elucidated. In this paper, we apply LDA, a mainstream topic model, to analyze the risk factor section from SEC filings (10-K and 10-Q), and describe risk structure change over the past two years. The results show that Covid-19 has transformed the risk structures U.S. companies face in the short run, exerting excessive stress on international interactions, operations, and supply chains. However, this shock has been waning since the second quarter of 2020. Our model shows that risk structure change (measured by topic distribution) from Covid-19 is a significant predictor of lower performance, but smaller companies tend to be stricken harder.

## Introduction

Covid-19 has transformed the U.S. business world. During the pandemic, companies have been forced to rethink their business models, adjust their employee organization, and better manage their supply chains. Covid has become such a significant event in our century that many people believe we have entered an era of a “new normal” [1]. However, as of now, we have yet to fully understood Covid’s profound influence on our economy and society, and the underlying mechanisms behind this process.

The impact of Covid-19 on firms is multi-dimensional. When Covid emerged, its most obvious impact was at the market and community levels. Supply chains were interrupted [2], and shocks ran through the stock markets of many countries [3, 4]. The structure of the labor market transformed [5], and how employees work fundamentally changed [6]. The early studies assumed that the economy and social structures were impacted by the pandemic as a whole. The pandemic shocks and reshapes the macro landscape, and the effect is transmitted to individual firms through invisible forces in the field.

However, scholars gradually notice that individual heterogeneity is important in the case of Covid. While the pandemic has become the last straw for some unfortunate companies in financial and management dead ends (for example, JC Penney, which went bankrupt during the pandemic), it has presented great opportunities for other firms. According to an article from the Brookings Institute, the United States’ leading retail and grocery companies, including Amazon and Walmart, have raked in billions during the Covid pandemic [7]. Explaining the variable performance is both possible and desirable.

Thus, some studies begin to focus on Covid’s direct effects on firms. Based on individual organization-level analysis, studies have shown that the pandemic has increased short-term financial pressure, especially for those who lack banking relationships [8]; hurt the supply chain, especially for those with insufficient technological inputs [9]; and disrupted stock returns and stakeholder attention, especially for those lacking input in corporate social responsibility [10]. The global crisis has a wide impact spanning across all industries. As this paper was written, we saw evidence of this from sectors such as manufacturing [11], exports [12], media [13], and construction [14].

These studies provide comprehensive details and useful perspectives, as well as many solid causal and correlation relationships. However, the industry-specific nature and survey methods of most previous studies have limited our ability to check the whole picture of risk structure dynamics in the time of Covid. As firms often need to balance objectives, it would be interesting to picture the time-varying structures of risks, evaluate their influence on company fundamentals, and draw a comparison between firms from different backgrounds. To achieve this goal and answer the question “How does Covid impacted the risk structure faced by U.S. public companies and influence their performance?”, we make use of the risk disclosures (item 1A) in public companies’ annual and quarterly reports, and generate the pattern of risk structure change by machine learning methods.

### Risk disclosures and their validity during a crisis

In December 2005, the SEC required registrants to discuss "the most significant factors that make the company risky" under the Risk Factors item (Item 1A) in their annual reports. This turned a voluntary discussion into an obligatory one. The policy has aroused wide interest in academia and industry. Besides the traditional theoretical interest in the validity of risk disclosure, researchers have also responded to investors’ curiosity about the timeliness and accuracy of risk disclosures, as many investors rely on annual and quarterly reports as the basis for their investing decisions.

Overall, evidence has shown that item 1A under the mandatory regime is a valid document which reflects practical risks, and can be used to make meaningful predictions. Nelson and Pritchard [15] found that while firms subject to greater litigation risk provide more comprehensive and timely disclosure under the voluntary regime, this difference disappeared after risk disclosure was mandated. This shows that even firms subject to lower litigation risk disclose risks more efficiently after 2005. Firms facing greater risk disclose more risk factors, and the type of risk a firm faces determines whether it devotes a greater portion of its disclosures towards describing that risk type [16]. Certain keywords in the risk factor section are related to changes in financial performance [17]. In sum, both the quantity and the structure of the risk disclosures reflect the external uncertainties.

Many of the studies about risk disclosure validity have focused on the crisis period, which is often the most chaotic time, when companies face high uncertainty in their environments. Fortunately, previous work has shown that risk disclosures do reveal the risk dynamics of a crisis, although only scant evidence has come from the U.S.

Crisis first leads to more comprehensive risk disclosures. A study based on Greek companies has shown that the quantity of risk disclosed goes up during a crisis; there was a significant increase in the instances of risk during the 2008 Financial Crisis [18]. Evidence from the U.K. has confirmed this conclusion, and also shown that the quality of disclosure during a crisis increases (i.e., the companies report more detailed and company-specific information) [19]. U.S. data shows that the risk factors do not appear to forecast future risks that have yet to be realized, but it does trace the accomplished facts in a timely way [20].

The studies mentioned above demonstrate that risk disclosure contains some objective and valid information about uncertainties, and that this information is updated promptly during a crisis. These corpora allow us to trace the change in U.S. public companies’ risk structures during the Covid-19 pandemic.

The total number of risk disclosures is quite large, and reading, coding, and interpreting them objectively in a limited time exceed human ability. For that reason, we involve computational topic modeling in this work. For processing the risk disclosure corpus, we train a Latent Dirichlet Allocation model (hereafter referred to as “LDA”), a mainstream topic model on the corpus, calculate the distribution of risk topics for each record, build measurements for Covid shock based on risk structure change, and test whether this shock has affected their performance. As all public companies in the U.S. follow the same standards for filing Security and Exchange Commission (hereafter referred to as “SEC”) documents, and are legally obligated to report their risks, descriptions across firms are comparable. As there are three quarterly reports and one annual report for most public companies each year, our data structure is longitudinal, which allows us to trace the change in companies’ risk factors across time.

### Hypothesis: The effects of Covid 19 on U.S. public firms

Previous studies have shown that Covid-19 has essential strikes on U.S. business. For individual companies, it has increased short-term financial pressure [8], hurt the supply chain [9], and disrupted stock returns and stakeholder attention [10]. Since the Covid pandemic has increased the uncertainties, and risk disclosure has been proved to be an honest reflection of external risks, hereby we put forward Hypothesis 1:

*H1: The risk shock related to Covid-19 will negatively affect U*.*S*. *public companies’ performance*.

In this study, we operationalize the firm performance as return on assets (ROA), which can also be factorized into net profit margin (NPM) and asset turnover ratio (AT). Thus, the following hypotheses can be deducted from H1:

*H1a: Covid related risk shock is negatively related to return on assets (ROA) during the pandemic*.
*H1b: Covid related risk shock is negatively related to net profit margin (NPM) during the pandemic*.
*H1c: Covid related risk shock is negatively related to asset turnover ratio (AT) during the pandemic*.

Large firms are traditionally believed to be more profitable [21], better-connected [22], and have more public policymaking influence [23]. Although all firms face external uncertainties in the macro-economic environment, small and medium-sized companies could be more vulnerable to the pandemic impact on average, as they don’t have the financial, research and network resources that large firms often have. This effect is particularly noticed during the Covid-19 pandemic, mostly through interviews, observations, and small-scale surveys [24]. Based on these facts, we have Hypothesis 2:

*H2: The negative relationship between Covid-related risk shock and U*.*S*. *public companies’ performance will be negatively moderated by firm size*.

## Methods

### Topic modelling in text analysis

With the growing availability of text data and computational power in the digital age, natural language processing (hereafter referred to as “NLP”) has increasingly been adapted in the social sciences. One of the most popular methods is topic modeling, a class of Bayesian probabilistic models that extract latent themes from a set of documents [25].

In a corpus, documents and words are often observed, but the logic for word combinations to form more complex semantic structures is latent. Topic modeling assumes the existence of “topics”, a “hidden structure” situated between individual words and documents. From a statistical perspective, documents are distributions of topics, and topics are distributions of words [26]. With data on the co-occurrence of words in documents, topic modeling aims to infer this latent structure. Thus, the outputs of topic modeling are the two distributions mentioned above. With the word distribution of each topic, we can label the semantic meanings for each topic, and summarize the most important themes for a corpus. With the topic distribution of each document, we can code the meaning composition for each document with little human labor.

Due to its power and efficiency, the topic modeling method has been used in various fields, including social and cultural analysis [27], finance [28], and management [29]. One of the practical applications of topic modeling is risk-type detection for SEC filings. The annual and quarterly reports of most U.S. public companies include a section titled "risk factors", usually in Section 1A. These sections disclose the uncertainties faced by the company at the time of reporting. Traditional methods in public companies’ risk detection usually rely on human coding to summarize risk types from a corpus [30]. With the rise of the computational age, machine learning methods, including KNN [17] and topic modeling [31, 32] have been adopted in automatic labeling and categorization. These studies have validated the accuracy of the algorithms, and a labeling system has been nearing maturity. Our study is built on these previous works, and incorporates a topic modeling and previously designed labeling system into Covid-19 risk dynamics.

In this paper, we use LDA algorithm, in which topic distributions for each document are drawn with a Dirichlet distribution [33]. LDA is one of the most popular algorithms in the topic modeling family, and also the most common one in the current management literature. Details of LDA algorithm can be found in S1 Appendix section 5.

### Data source and research design

Our corpus comes from the 10-K and 10-Q filings from the U.S. Securities and Exchange commission (SEC) during 2020–2021. The 10-K is a comprehensive report filed annually by a publicly traded company about its major business, management conditions, and financial performance. The 10-Q is the quarterly report providing similar information. We first collected a list of current active public companies in the U.S. from the SEC website, then used this list to scrape 10-K and 10-Q text. In most cases, companies provide one annual report and three quarterly reports per year (one during each financial quarter), although the specific dates of publications may vary slightly. We collected this data in October 2021. Since the first case of Covid-19 was reported in the U.S. in January 2020, each company should have provided around seven risk factor records. Some quarterly reports do not add new information based on the 10-K annual report (For example, in their March 2021 10-Q report, Apple states: “…There have been no material changes to the company’s risk factors since the 2020 Form 10-K”); in these cases, the information cited will be replaced by the 10-K risk factors in the referred 10-K. If a 10-Q report provides only one or two short sentences (e.g., “none”) that do not cite another report, we would delete these records. In total, we collected 28,981 valid records for 4,404 listed firms, with some records missing due to length or quality issues.

Although risk factors (Item 1A) in the SEC filings are often regarded as the products of investor impression management, previous studies have shown that this section traces and reflects temporary risk change during a crisis [20], and contains information on risk structures that can be used in meaningful predictions [17, 34]. The structure of the risk disclosure document is also reliable [16]. Thus, we believe measurements based on this corpus will be valid.

Following previous work on risk factor extraction, we trained an LDA model to extract risk topics from text in the corpus, and calculate the risk distributions [31]. For training LDA model, we use Gensim package in Python.

The parameter choices generally have immense influence on the model’s quality, especially regarding the number of topics. For this reason, we tried seven possible quantities of topics, from 30 to 100, at every multiple of 10. We chose 30 as the starting point because previous work on risk factor identification [17] has summarized 25 topics based on industrial experience, and we wanted our model to be fine-grained enough to be able to include each of the topics. Many previous studies involving human labeling as a downstream process limit the number of topics to 100, to ensure that topics are interpretable for human coders [35]. Adapting this consideration, we chose 100 as the endpoint.

**Fig 1:**
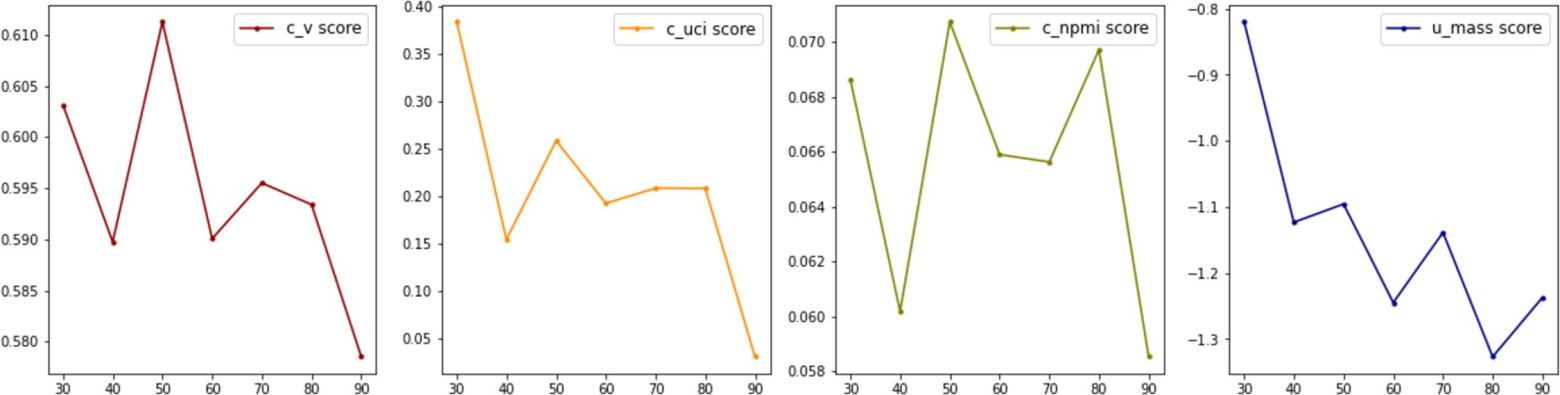
Coherence scores of LDA with topic number 30–100.

After training all the models, we calculated the coherence score for each model. A coherence score measures the extent to which all of the words in one topic are close to, and in support of, each other; a higher coherence score is a sign that a model has good fitIn general, there are four possible algorithms for calculating coherence (c_v, u_mass, c_uci, c_npmi). The results are shown in Fig 1. Based on the results of the coherence scores, models with 30 topics produce highest c_uci and u_mass score, while models with 50 topics produce highest c_v score and c_npmi score. Thus, we may safely conclude that 30 and 50 are the two optimal topic quantities, depending on which metrics we use. To reduce information loss, we adopted the more fine-grained 50-topic model. Thus, all of the ensuing results are based on LDA50.

**Fig 2:**
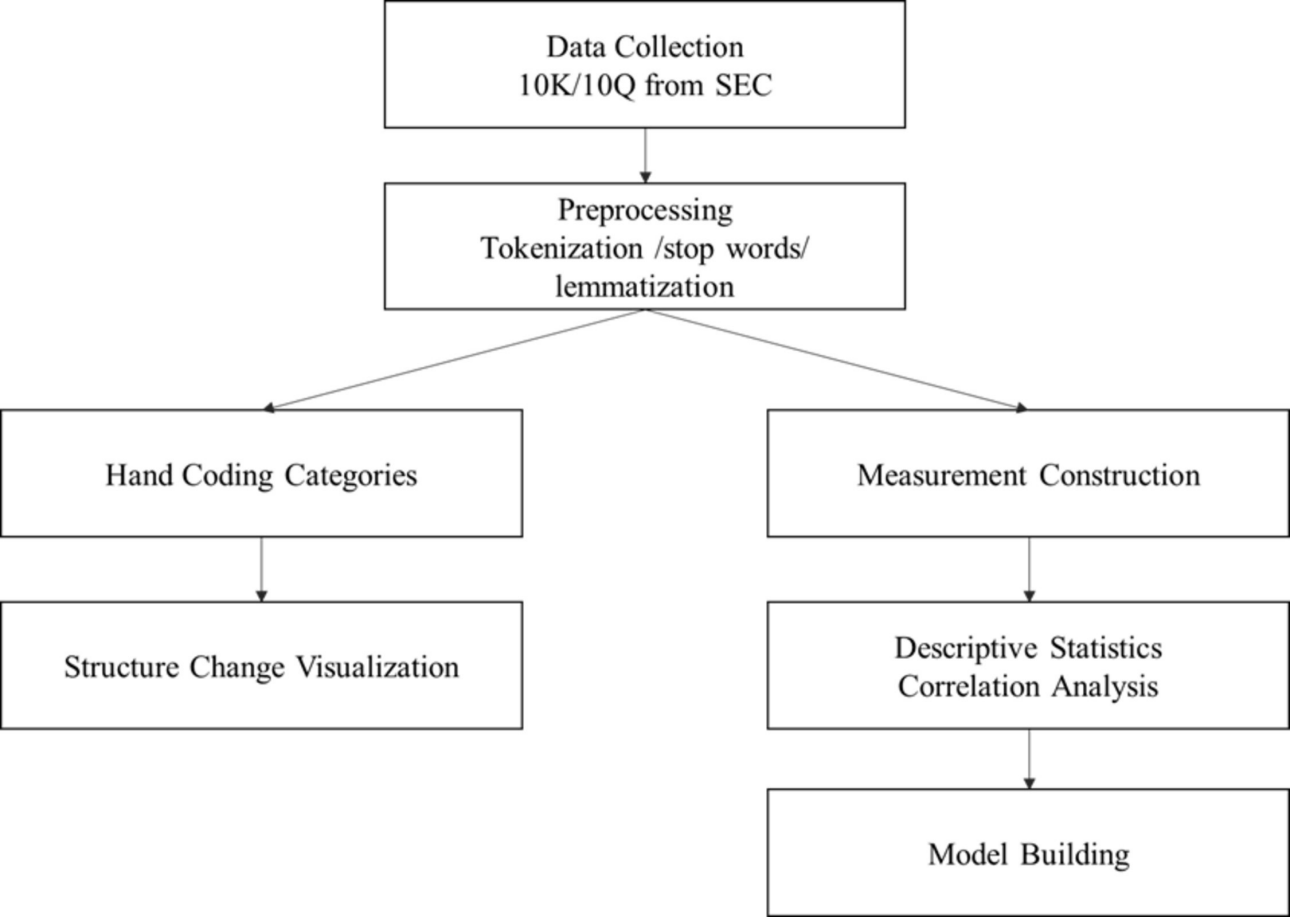
Flow chart of methodology.

After calculating the distribution of risk topics for each document, we present our results in two ways. First, we hand-code the risk topics, collapse them into several small categories, and describe the change in risk structures over time. Second, we calculate two measurements for Covid risk shock based on the original 50-topic risk distribution, and use them in the model to test the effect of the Covid-19 pandemic on U.S. public companies’ performance. The flow chart for our methodology is shown in Fig 2, which shows the pipeline of our methods.

For the first part of the analysis, we hand-coded each topic, based on the categorization system put forward by Huang and Li [17]. This system is well-designed and has been adopted in risk factor identification studies [31]. Two researchers hand-coded the topics independently and matched their coding afterwards; the agreement rate was 0.94, with only three topics coded differently. After discussion, we would either reach an agreement, or code the disputed topic as “other”, as this disagreement shows some ambiguity. It should be noted that this hand-coding process only influenced the visualization results. We calculated the independent variables in the panel analysis based on the original topic distribution, which was not influenced by a subjective factor.

As representative words sometimes have ambiguous meanings that can be used for two topics (for example, “patent” could be a word for intellectual property risk, or lawsuit risk; “loss” could be related to financial condition risk or shareholder interest risk), we collapse the semantically close risk topics into 12 large categories. The smaller number also helps us to create simple and clear visualizations. For the second part, we calculate the measurements for Covid-19 risk shock, as shown below.

### Measuring Covid-19 related risk shock

Previous work has shown that crises often lead to shifts in companies’ risk distribution. This leads to increases in certain types of risk factors and increased details in the report [18, 19]. Thus, the change in risk structure serves as a sign of shock. Based on these insights and our topic model, we built two indicators to measure the intensity of Covid-19 on risks listed firms encounter, i.e., risk structure shock, and risk structure dispersion.

#### Risk structure shock

We first extracted Covid-19 related sentences (any sentence containing the words “Covid”, “coronavirus”, “pandemic” or “epidemic”) from each record, and calculated each company’s Covid-related risk distribution based on our topic model. We measured risk structure shock with the Hellinger distance between a company’s risk topic distribution for a certain quarter and the company’s overall risk distribution in 2020 Q1. We used 2020 Q1 as the baseline, because the first case of Covid-19 in the U.S. was detected on January 21, and it had attracted little business attention by that point (The rare mention of Covid-19 in the risk factors, as shown in Fig 2 Panel 3, provides some support for this argument). A greater Hellinger distance between two distributions means that Covid-19 has transformed the risk structure the company has encountered, and has brought many unfamiliar risks which the company might not have otherwise faced.

#### Risk structure dispersion

After we calculated the Covid-related risk distribution, we used the negative value of Herfindahl-Hirschman Index (HHI) to measure the extent to which Covid-related risks were dispersed across multiple aspects of business operations. The Herfindahl-Hirschman index is widely used in economics and sociological studies to measure the degree of concentration [36], and negative value of Herfindahl-Hirschman index measures the contrary, i.e., how dispersed the risks related to Covid-19 have been. To factor baseline risk concentration level into consideration, we construct this measurement as:

$$risk\;structure\;dispersion\;\left(for\;firm\;i\;at\;time\;t\right)=-\frac{covid\;HHI_{it}}{baseline\;HHI_{i1}}=-\frac{\sum p_{cit}^{2}}{\sum p_{oi1}^{2}}$$

A higher value for this variable is a signal that Covid has broadly impacted multiple aspects of the company, and the extent of risk dispersion has exceeded a company’s familiar level.

After constructing the measurements, we carry out panel data analysis. We fit both the random-effects and fixed-effects models to the data. The fixed effects model has the form:

Yit=β*Xit+αi+uit,fort=1,2,…Tandi=1,2,….N.

And the random effects model has the form:

Yit=β0+β*Xit+αi+uit,fort=1,2,…Tandi=1,2,….N.

Where i refers to the index of individual companies, t refers to the period index in the data, *Yit* refers to the dependent variable of company i at time t, and *Xit* refers to the independent variables of company i at time t, including covid-related risk measures and controls. Details of Panel data analysis can be found in S1 Appendix section 5.

### Other variables in the random and fixed effects models

In the panel data analysis, our dependent variables and controls are:

#### Dependent variable

Following previous studies, we use return on asset (hereafter referred to as “ROA”), a common financial ratio, to measure company performance [37, 38]. We also adopt the frame of DuPont Analysis in which ROA is a product of net profit margin (NPM) and asset turnover ratio (AT), and regress it on these two variables to test the path of Covid impact.

Previous studies [39–41] have found that disclosures stimulate market responses. Following these precedents, we also used cumulative abnormal return (hereafter referred to as “CAR”) to measure the market response. The models with CAR as dependent variables are reported in Table S1 in S1 Appendix section 3.

#### Controls

In our panel data model, we controlled: (1) text features, including the length of Covid-related risk statements, and the proportion of Covid-related text throughout the risk factor text; (2) category of major risk (dummies); (3) firm financial and operational measurements, including quarterly total assets (with log scale), debt-to-asset ratio, and R&D intensity; (4) company background information, including location (state dummies) and industry (SIC class dummies). In the section about the network buffering effect (S1 Appendix section 4), we also used degree centrality and clustering coefficients in the control variables.

Next, we collected the quarterly financial ratios and fundamentals for the companies from Compustat, a popular dataset that traces the dynamics of U.S. listed firms. As of the completion of this paper, Compustat quarterly financial ratios had been updated to 2020 Q4. Therefore, we only use four financial quarters’ data in our models.

The panel data analysis is completed with Stata.

## Results

### Time-varying risk factors related to Covid-19: Shock and recovery

**Fig 3:**
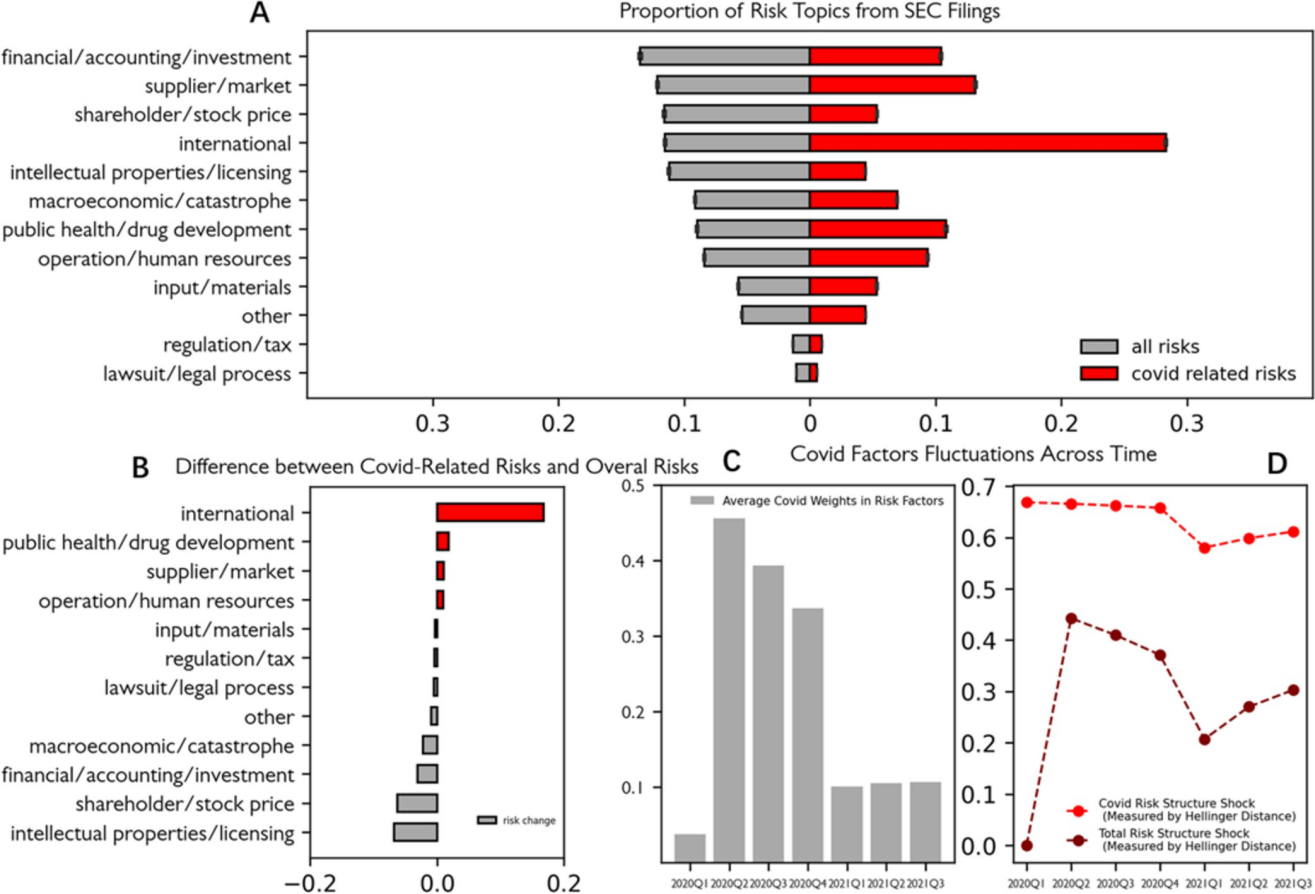
Distribution of risk topics during 2020 Q1 and 2021 Q3.

LDA allows us to calculate risk distributions from documents. An average risk distribution for U.S. companies from 2020 Q1 to 2021 Q3 is shown in Fig 3 Panel A (the proportion of each risk types in all corpus vs. covid-related corpus), and the difference between Covid-related risk distribution and the overall risk distribution can be found in Fig 3 Panel B (the difference between two proportions mentioned above). The results show that one of the most profound effects of Covid comes from international risks, including restricted transportation, currency exchange rate volatility, and cross-border business interactions. In addition, Covid has stressed U.S. public companies’ supply chains and operations. In a time of crisis, other risks, such as regulation, accounting, shareholder interests, and lawsuits fade temporarily from people’s attention.

Panel C of Fig 3 shows the average proportion of Covid-related sentences throughout the entire risk factor corpus, from 2020 Q1 to 2021 Q3. The proportion is calculated by first dividing the number of covid-related sentences by the total number of sentences in the risk statements for each company, and then average the score for all companies in the quarter. The data indicate that the U.S. business world was shocked by Covid-19 in the second quarter of 2020; attention soars from less than 0.1 to nearly half of the space in the risk section. A slow recovery began in the third quarter of 2020, until there was a sudden reduction in 2021 Q1.

Panel D of Fig 3 shows a similar pattern. In Pattern D, we measure the extent of risk structure shock from Covid-19 via the Hellinger distance between a company’s risk distribution for a given quarter and its overall risk distribution in 2020 Q1, as we have explained in the measurement construction section. Here, for the company’s risk distribution in a given quarter, we try both overall risk distribution, and Covid-related text risk distribution. The patterns are similar. We see slow recovery since 2020 Q3, and a sharp recovery in 2021 Q1. The Covid effect continues growing a bit afterward.

**Fig 4:**
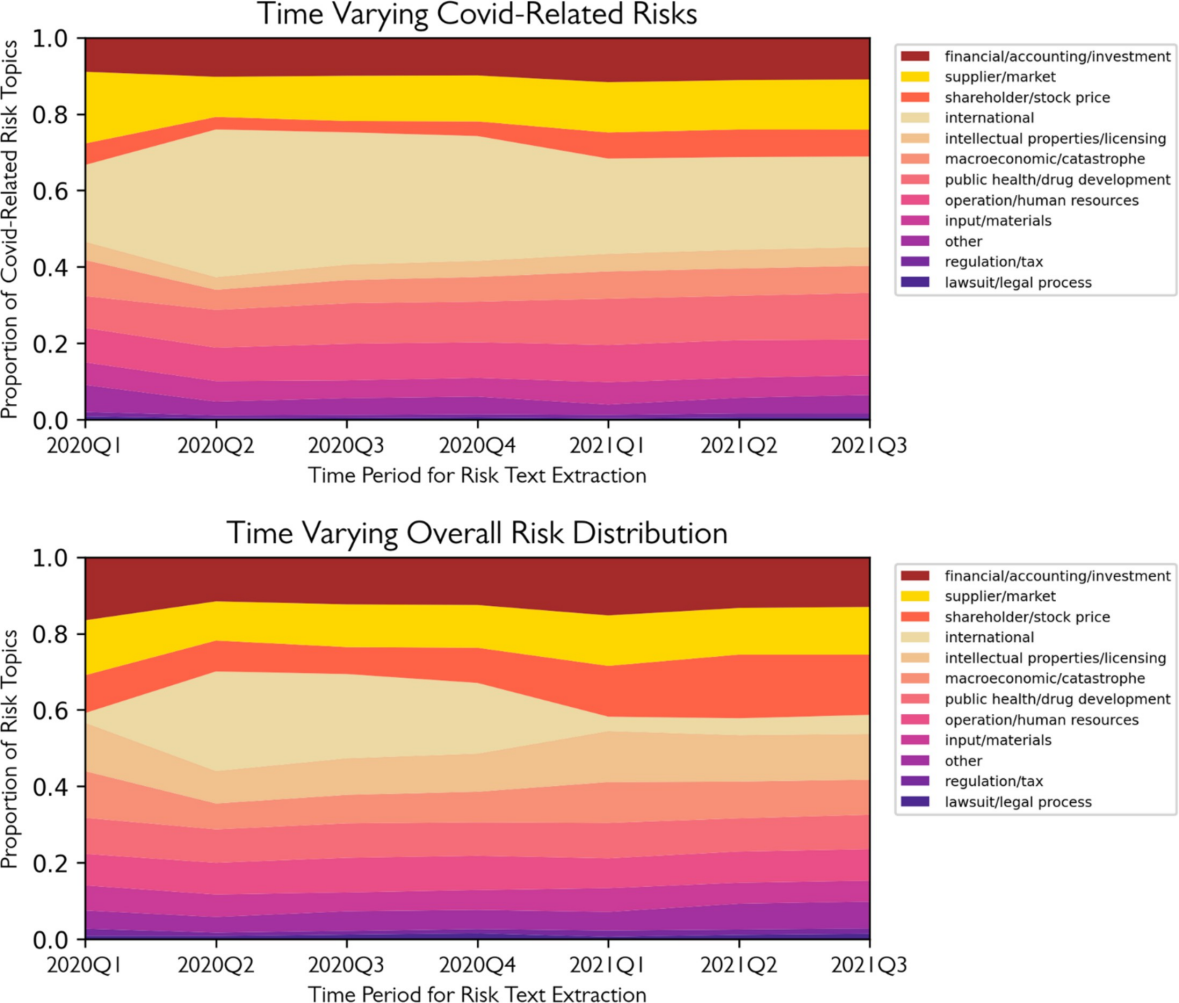
Risk structure change across time.

Fig 4 shows the time-varying overall risk distributions for U.S. public companies. As we get the proportion of each risk type from the topic model for each period, which adds to 1, here we present them in a stack plot. Consistent with our other findings, there is an expansion of international risk in 2020 Q2, and a slow recovery afterward. By 2021 Q3, the risk structure U.S. public companies faced had recovered to the pre-Covid level. Although the global transportation and interactions have not fully resumed in 2021 Q3, U.S. companies appear to have found a way to address the situation. The average risk structure stabilizes after 2021 Q1.

As different industries often face different risk structures, we also conducted industry-specific risk distribution analysis. The results are presented in Fig S1 in S1 Appendix.

### Inequality of risk exposure during the pandemic

**Fig 5:**
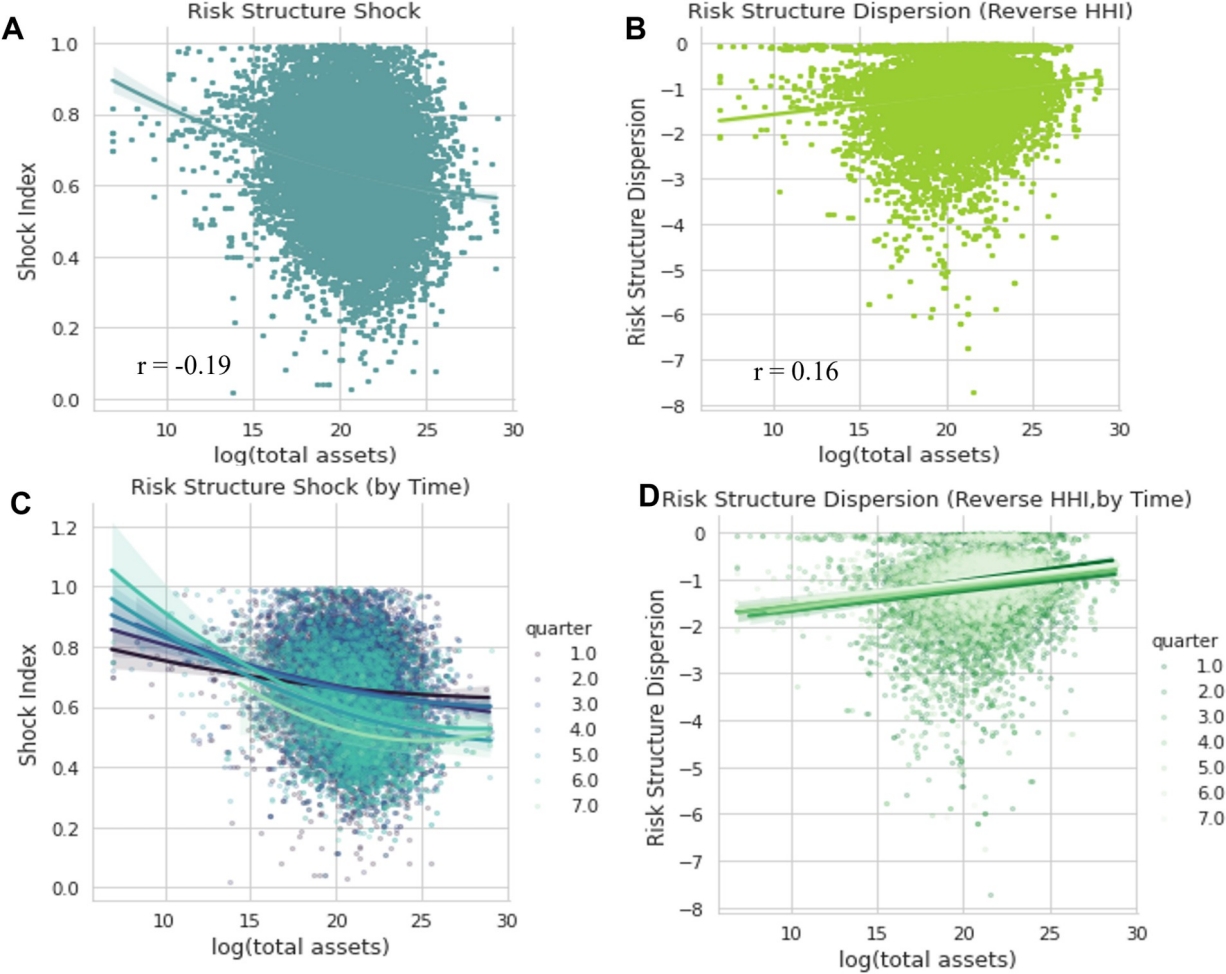
Relationship between risk shock measurements and firm size.

It is often assumed that small and medium-sized companies are more vulnerable to the pandemic impact [24]. In Fig 5, we use scatter plots and their regression fit to assess whether there is a relationship between firm size and our two measurements of Covid risk shock. We use log (total assets) to measure firm size, following previous studies [42], as the total asset distribution is skewed. Our two measurements for risk shock are on the Y-axis. The figures are generated by Seaborn package in Python.

Our data visualization partly supports the inequality hypothesis. For our two measurements, Covid structure shock is negatively correlated with firm size, as is shown in Panels A and B in Fig 5. This inequality appears to grow over time. From 2020 Q1 to 2021 Q2 (i.e., Quarters 1–7), the value of the negative correlation between firm size and Covid risk structure change increased, as is shown in Panels C. However, we also notice that Covid-19 tends to impact large companies in more aspects.

Thus, in the later stages of the Covid era, although in general, the effect of Covid appears to have decreased, small- and mid-sized public companies still face many unfamiliar risks that they might not have dealt with in the past, and these risks tend to be slightly more concentrated. In comparison, larger firms tend to recover faster.

Prestigious as they are as a group, public companies still face in-group inequality, and smaller organizations are more vulnerable under public crisis. Two short case examples are presented in S1 Appendix section 2.

### The covid effects on company performance

Following previous work, we use ROA as the indicator for public companies’ performance [37, 38]. As our data has a panel structure, we fit both random effects and fixed effects models. Random effects models are shown in Table 2, while fixed effects models are shown in Table 3.

Major variables’ descriptive statistics and correlation coefficients are reported in Table 1.

**Table 1:** Descriptive statistics and correlation table of major variables.

	MEAN	STD	1	2	3	4	5	6	7	8
(1) ROA	-0.014	0.302	1							
(2) Covid-related risk structure shock	0.652	0.162	-0.1074*	1						
(3) Covid-related risk structure dispersion	-1.142	0.746	0.0443*	-0.1357*	1					
(4) Covid text length	20.613	21.857	-0.1272*	-0.3931*	-0.0453*	1				
(5) Proportion of covid related text	0.251	0.263	0.1303*	0.1108*	-0.1630*	-0.0232*	1			
(6) R&D Intensity	0.019	0.050	-0.6917*	0.0655*	-0.00750	-0.00230	-0.00800	1		
(7) Debt to asset ratio	0.617	0.304	0.1104*	-0.0629*	0.0586*	-0.0587*	0.1268*	-0.1004*	1	
(8) Firm Size	21.091	2.149	0.4765*	-0.1734*	0.1545*	0.0189*	0.1164*	-0.0611*	0.3186*	1

*Note*: * *p* < 0.05.

The random effects models are reported in Table 2. Model 1 shows that only risk structure shock predicts company performance decline significantly, while risk structure dispersion has an insignificant coefficient (coefficient = -0.029*** for risk structure shock, and 0.002 for risk structure dispersion). This shows that Covid-related risk structure shock does influence company fundamentals. When a firm faces new and unfamiliar risks due to Covid-19, their performance tends to decline. However, the insignificant coefficient for risk structure dispersion shows that Covid-19 does not impact companies by narrowing the risk scopes.

**Table 2:** Random effect model of covid-19 risk shock on company performance.

	Model 1	Model 2	Model 3	Model 4	Model 5	Model 6
	Return on Assets	Return on Assets	Asset Turnover Ratio	Asset Turnover Ratio	Net Profit Margin	Net Profit Margin
Covid-related risk structure shock	-0.029*	-0.033**	-0.048**	-0.049**	-4.748	-5.407
Covid-related risk structure dispersion	0.002	0.001	0.005	0.005	-2.463+	-2.107
Covid text length	-0.000+	-0.000+	-0.001***	-0.001***	-0.065	-0.064
Proportion of covid related text	-0.007	-0.006	-0.059***	-0.058***	4.752+	5.168+
R&D Intensity	-1.011***	-1.013***	-0.049	-0.046	110.000***	110.000***
Debt to asset ratio	-0.077***	-0.076***	0.033	0.033	2.205	2.127
Firm Size	0.065***	0.064***	-0.046***	-0.046***	4.855***	4.963***
Risk structure shock * firm size		0.005**		0.001		0.112
Risk structure dispersion * firm size		-0.001		-0.001		2.366**
Major risk category (dummies)	Controlled					
Industry (SIC code, dummies)	Controlled					
Location: state (dummies)	Controlled					
_cons	-1.195***	-1.178***	1.470*	1.475*	-97.060	-99.890
*N*	8288	8288	8288	8288	8288	8288
R2 (overall)	0.431	0.435	0.296	0.296	0.003	0.004

*Note*: ^+ ^*p* < 0.1,

* *p* < 0.05,

** *p* < 0.01,

*** *p* < 0.001.

In Model 2, we add the interactive terms between firm size and Covid risk measurements. It appears as if Covid-19 has a dampened effect for large companies, with a significant positive coefficient (coefficient = 0.005, p<0.01) for the interactive term between risk structure shock and firm size. The interactive term between risk structure dispersion and firm size is not significant for return on assets (ROA) model; however, the interaction coefficient is significant for model 6 (coefficient = 2.366, p <0.01), showing that firm size buffer the negative effect of risk structure dispersion on net profit margin. Thus, the inequality of Covid-19 shock exists in two senses: (1) smaller companies face higher risk of structural shock, which means they face more unfamiliar risks; (2) even under the same risk structural shock, the smaller companies suffer more, and this is reflected in their performance.

As ROA is calculated as the product of net profit margin (NPM) and asset turnover ratio (AT), we regress our independent variables on these two variables to test the path of the causal effect. The results are shown in Models 3–6 in Table 2.

The models above show that Covid-related risk structure shock has affected U.S. public companies primarily by slowing asset turnover (coefficient = -0.048***). However, on average, it has not harmed net profit margin, with only Covid related risk structure dispersion having a marginal effect (coefficient = -2.463, p<0.1). Rather, the variation across companies appears to be primarily due to an interrupted supply chain.

Fixed effects models in Table 3 show a slightly different story. For individual companies, distortion of risk structure due to Covid-19 across time tends to decrease the asset turnover ratio (coefficient = -0.052, p<0.05), while wide dispersion of Covid effects across multiple aspects of organizational operations decreases the net profit margin (coefficient = -9.031, p<0.001).

**Table 3:** Fixed effect model of covid-19 risk shock on company performance.

	Model 7	Model 8	Model 9
	Return on Assets	Asset Turnover Ratio	Net Profit Margin
Covid-related risk structure shock	-0.012	-0.052*	9.218
Covid-related risk structure dispersion	0.000	0.001	-9.031***
Covid text length	0.000	-0.000	0.084
Proportion of covid related text	0.010	-0.000	-0.352
R&D Intensity	-0.061	-0.118*	134.900***
Debt to asset ratio	-0.252***	-0.113**	12.530
Firm Size	0.058***	-0.117***	-3.721
Major risk category (dummies)	Controlled		
_cons	-1.070***	3.274***	26.200
*N*	6137	6137	6137
R2 (overall)	0.170	0.021	0.008

*Note*: ^+ ^*p* < 0.1,

* *p* < 0.05,

** *p* < 0.01,

*** *p* < 0.001.

To summarize, the risk shock from Covid-19 does harm the fundamentals of U.S. public companies, while large firms tend to be impacted less. How does the inequality emerge? One possible mechanism is the social network buffering effect in the business world, as predicted by the network school of sociology [43]. We explore this possible mechanism in section 4 of the S1 Appendix.

## Conclusion and discussion

### Unequal vulnerability in the age of Covid-19

Covid-19 has transformed the lives and opportunities of thousands of public companies. In this paper, data visualization based on the topic model shows that Covid-19 exerts excessive pressure on public companies by interrupting international interactions, operations, and supply chains. With the end of the pandemic and the recovery of global transportation, many expect that the business world will soon recover to pre-Covid times.

However, our study reveals one of the challenges policymakers face: public companies in the U.S. have been impacted unevenly by Covid-19, and this discrepancy has not abated over time. Smaller companies generally face more unfamiliar risk categories that they might not have encountered in the past. This vulnerability may partly be attributed to the lack of, or disadvantaged positions within, respective business social networks, which often function as safety nets [44, 45] (for analysis, see S1 Appendix section 4). Although it appears as if the average risk composition has recovered to pre-Covid levels, the dynamics of underlying social structures reveals another side of the story: the Matthew effect is present, and may permanently change some parts of the business landscape, even when we begin to expect the ending of Covid-19.

This research only includes U.S. public companies, which are typically considered a prestigious group in the business world. If the mechanisms and dynamics in our work hold true, small/mid-sized enterprises should be in even worse shape. Organizational analysis rarely adopts the inequality perspective. Yet as in other social sphere, business organizations are also constrained by unequal opportunities in social structures.

### Divergence between fact and expression

Machine learning can extract complex patterns and dynamics from corpora, but can a human reader derive intuitive impressions about the Covid impact from the risk factor section of an annual/quarterly report? To explore this question, we check several expressions’ variables, including the proportion of Covid-related text in the risk factor section, and the negative sentiment score calculated from covid-related sentences.

For sentiment score calculation, we use a traditional method based on positive/negative word count. For each document to be evaluated, this method first counts the number of positive words and negative words, respectively, based on a priori dictionaries, then calculate the sentiment of the document as:


Sentimentscore=(positivewordcounts-negativewordcounts)/totalwordcounts

Following Loughran and McDonald’s [46] suggestion, we only count negative words for financial filing sentiment analysis, as positive emotions in financial statements are not very obvious. We use their dictionary that targets financial documents as the basis for sentiment score calculation. The resulting negative score = negative word counts/total word counts.

**Fig 6:**
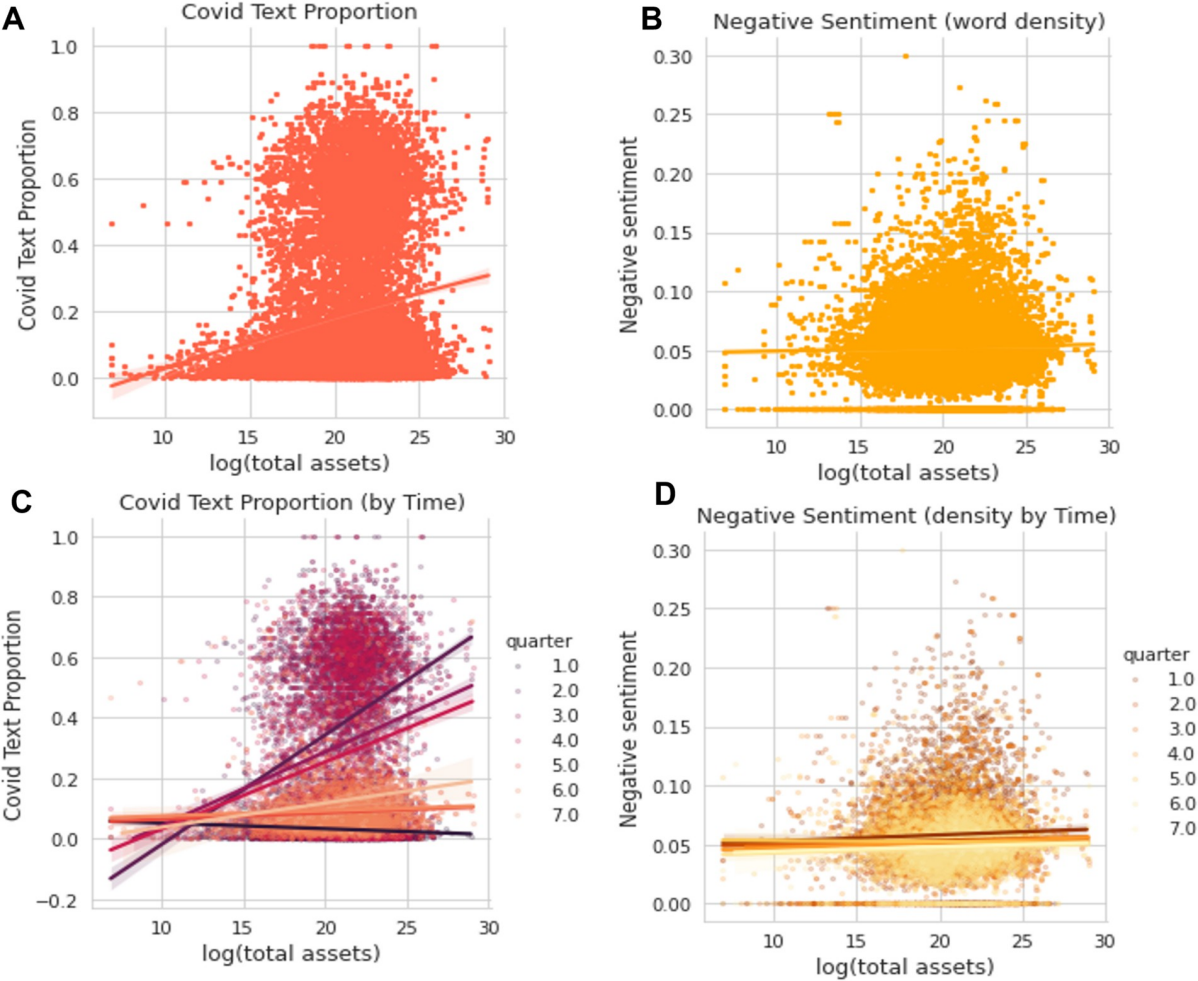
Relationship between covid-related expressions and firm size.

The results reveal a contradiction—larger companies typically talk more about Covid-19 (correlation coefficient = 0.16) and appear to be slightly more negative (correlation coefficient = 0.02, a small positive value), however they are not necessarily impacted harder (Fig 6). This pattern is not due to the length of the 10-K or 10-Q risk factor text (we measure the length of the risk text and correlate it with firm size, but only get a negative correlation coefficient of -0.0972).

Time-varying facets also show larger companies to be more sensitive to the environment—in 2020 Q2, when the Covid-19 impact on business first emerged, they reported Covid-19 risk with longer text. This discrepancy in reporting style disappears after 2021 Q1. However, this discrepancy had been in place throughout the more turmoil 2020 Q2-Q4. Although the negative correlation between firm size and negative sentiment is only marginal, it stabilizes across the seven quarters we test.

In sum, larger public companies are affected less, but talk more. Based on the risk reporting, they do not appear optimistic about the pandemic; in reality, they appear a little more pessimistic, although the effect is marginal and decreases with time. Although we can regard the divergence between reality and business expressions in the SEC filings as a signal of quicker risk detection and more timely response to the external uncertainties for the larger firms, we remind researchers and investors to note this divergence. Future studies could expand on this exploration to assess whether larger firms are superior at risk management, or just more experienced in investor relations maintenance.

### Utilizing the text analysis to understand social processes

Our work is one of the earliest systematic, longitudinal studies of the Covid-19 effect across all industries. Limited by sample size and survey methods, previous work on covid-19 shock often focuses on one aspect of risks and one specific industry. In this study, we compare the risk structure change between firms from different backgrounds across several quarters. We also contribute to the crisis literature by revealing the inequality of vulnerability among firms. Although all firms have been shocked by the same event, their social structures and social network positions will largely influence their reactions and outcomes induced by the shock. From a practical perspective, our work calls on policymakers to pay more attention to the marginal players in the market.

Managerial teams can use our work as a reference to evaluate the effects of the Covid pandemic on their organizations. Although Covid shocks all organizations, their effects are unevenly distributed across industries and periods. Long-lasting distortion of risk structures can be an alarming signal, as they are related to both lower performance, and worse competitiveness in a market where more powerful competitors recover faster. Under the current regime, Covid might have permanently changed U.S. economic landscape.

From a methodological perspective, this study is a trial of the application of a well-developed NLP technique on a temporal, longitudinal corpus. We combine social science and algorithms to describe the underlying social dynamics. Text is often fine-grained and multi-dimensional, with meanings and symbols entangled in complex semantic structures, and these meanings are often fluid over time. Text analyses allow us to trace these subtle changes (or unchanged meanings) in a quantitative frame. The social events that take place in the real world, cultural expressions and constructions that emerge in the symbolic domain, and the interactions and co-evolution between these two spheres [47], constitute a more comprehensive picture of the story, and allow for a dynamic understanding of the social process.

## Supporting information

S1 Appendix This document presents additional analysis and case examples related to the analysis in the main text. (DOCX)

S1 File (DOCX)

S2 File (ZIP)
