# Supplementary material for: Machine learning shows that the Covid-19 pandemic is impacting U.S. public companies unequally by changing risk structures
Source: PLoS One. 2022 Jun 22;17(6):e0269582. doi: 10.1371/journal.pone.0269582 (PMC9216502; doi:10.1371/journal.pone.0269582)
Supplement: S1 File — (DOCX) [file pone.0269582.s002.docx]

## **S1 Fig. Risk Structure Change Across Time (by Industries)**

We replicate the analysis in Fig 3 to track the industry-specific patterns in risk change related to Covid-19. In this fig, the industrial categories are based on SIC codes. We can see that Covid-19 has brought excessive international risk to most industries, but some interesting trends may need more attention. For example, Covid has introduced financial/accounting pressure into the construction industry, and this trend has yet to abate until 2021 Q3. From 2020 Q1 onward, the finance, insurance and real estate industries have reported higher shareholder risk and stock prices, and this trend has continued. Simple as they are, these figures may provide signals of what is transpiring in the market.

## **S2 Appendix. Case Examples**

To present our arguments and measurements more intuitively, we use two public companies as cases to demonstrate the Covid-19 shock over time. The first company is Walmart Inc. (ticker: WMT), one of the most famous multinational retail corporations based in the United States. They are the operator of a chain of hypermarkets, discount department stores, and grocery stores. The second is JCPenney (ticker: JCP), a mid-scale American department store chain with 689 locations in the United States and Puerto Rico. During the pandemic, JCPenney filed for Chapter 11.

For a more comprehensive understanding of risk structure evolution, we hand-collected the two case companies’ risk factor text, from 2019 Q3 to the latest record (for WMT, the endpoint was 2021 Q3; for JCP, the endpoint was 2020 Q4, the last filing record on the SEC website). After obtaining the risk text, we calculated the risk distributions for each time point and visualized the risk structure change during 2019-2021. The results are shown in Fig below.

**S2 Fig. Risk Structure Change for Two Cases**

The impact of Covid-19 on Walmart has been slow and gentle. At the beginning of 2020, there was a small increase in supplier/market risk, but the risk structure is not radically different from that of 2019. In 2021, the risk structure diversified a bit, including some shareholder/stock price risks, international risks, and IP/licensing risks which had not previously appeared. However, in general, the risk shock is incremental, and there is the prospect of recovery. In fact, based on an article from the Brookings Institute, Walmart has earned about 5 billion dollars in additional profits compared with the same period in 2019, showing a reverse Covid-19 effect [7].

In comparison, JCP has not been so lucky. JC Penney is a department store with multiple headquarters. Before Covid-19, it had faced fierce competition from retailers such as Walmart, Macy’s and Target, and had not reported a profit since 2010. In 2020 Q1, JCP was in a disadvantaged financial situation (for example, in their risk factor disclosure, they claimed, “our level of indebtedness may adversely affect our business and results of operations and may require the use of our available cash resources to meet repayment obligations, which could reduce the cash available for other purposes.” … “Operating results and cash flows may cause us to incur asset impairment charges.”). Shareholder interest is at stake as well (“If we cannot meet the continued listing requirements of the NYSE, the NYSE may delist our common stock.”). In 2020 Q2, Covid-19 was the last straw—JCP’s debtors commenced Chapter 11 filings, marking the beginning of the bankruptcy reorganization process. This legal process was repeatedly mentioned in the risk factors during 2020 Q1-Q4, the final year when JCP acted as a public company. As shown in Fig S2, Covid-19 has elicited radical change in JCP’s risk structure, culminating in its collapse.

## **S3 Table. The Effect of the Covid Risk Shock on Market Response**

|  | Model S1 | Model S2 |
| --- | --- | --- |
|  | CAR | CAR |
| Covid-related risk structure shock |  | 0.009 |
| Covid-related risk structure dispersion |  | 0.004 |
| Covid text length | 0.001^***^ | 0.001^***^ |
| Proportion of covid related text | 0.050^***^ | 0.052^***^ |
| R&D Intensity | 0.070 | 0.066 |
| Debt to asset ratio | 0.001 | 0.002 |
| Firm Size | -0.001 | -0.001 |
| Major risk category (dummies) | Controlled | |
| Industry (SIC code, dummies) | Controlled | |
| Location: state (dummies) | Controlled | |
| _cons | -0.201 | -0.199 |
| *N* | 8208 | 8208 |
| R2 (overall) | 0.021 | 0.021 |

*Note:* ^+^ *p* < 0.1, ^*^ *p* < 0.05, ^**^ *p* < 0.01, ^***^ *p* < 0.001.

To test market response to the Covid shock, we regress the same set of variables from Table 2 on CAR. The models are reported in Table S3.

For all independent variables and controls, our measurements for Covid-related risk shock have no significant effect on market response, although they do predict lower performance in Table 2. In comparison, the companies that talk more about Covid-19 in the risk factor section tend to receive better evaluations from investors (both Covid text length and Covid text proportion have significant, positive coefficients). This might be attributed to the investors’ tendency to trust more candid reports, but this subtle psychological process lies outside the scope of this work. The results in Table S3 also partly explain large firms’ motivation to talk more about Covid in their reports.

## **S4 Appendix: The Social Network Buffering Effect**

In the business world, social networks act as signals for status, and pipelines for resource transmission [48]. For the opportunities, resources, and social capital that come with them, networks often buffer external risk as a safety net [44-45]. Research has shown that this function may be more salient in situations where formal institutions are unavailable, or where the external environment is unstable, as social networks can generate otherwise scare trust and social norms that support valid social cooperation [49].

During the Covid-19 crisis, social routines have been interrupted. As such, social networks can plug the breakage, establishing new ties in the supply chain. Two mechanisms, information and control, both lead to this effect. On one side, network connection helps a company know more potential customers and suppliers. This allows them to replace the old ones once a relationship has been broken. On the other side, when facing competitors, network advantage allows well-connected companies more opportunities: either to slow the loss of original orders, or to secure new orders quicker. Thus, the network effect should be one of the potential mechanisms leading to business vulnerability inequality in the wake of the pandemic.

We first obtained data on 2019-2020 directors from Compustat (the most recent updates as of the completion of this paper), and generated a public company interlocking director network on this basis. As an essential part of the business community, the interlocking director network is one of the most well-studied inter-company networks; it brings immense rewards to those who occupy advantageous positions [50-52]. Previous studies have shown that this network systematically improves companies’ performance, especially in uncertain environments [45]. For our study, the 2019-2020 network is visualized in Fig S4A.

**S4A Fig. Interlocking Directorate Network for S&P 1500 during 2019-2020**

**(Left: colored with degree centrality; Right: colored with covid structure shock)**

In the upper panel of Fig S4A, we color the nodes by degree of centrality, one of the most common ways to visualize networks in similar studies. For the lower panel, the size and color of the nodes indicate the Covid shock measurement (Covid-related risk structure shock). In both figures, nodes with greater values on the two scales are larger and darker. Comparing the two panels reveals a pattern: some of the most heavily affected companies are on the periphery of the network, while the inner circle tends to be better protected. We statistically test this hypothesis later in this section.

To calculate an individual company’s positional advantage within the network, we adopt two of the most prevalent measurements in social network analysis: degree centrality, and clustering coefficient [43]. Degree centrality measures how many ties a node has in a network. Higher centrality indicates that the actor is well connected, and is often the sign of high status [53] in a field. The clustering coefficient is defined as:

$C_{i}=\frac{2|\{e_{jk}:v_{j},v_{k}\in N_{i}, e_{jk}\in E\}}{k_{i}(k_{i}-1)}$ (2)

This measures the proportion of realized ties among one’s ties in their neighbor community. Compared with degree centrality, which is a global measurement, the clustering coefficient serves more as a local measurement. A high value for the clustering coefficient shows that a social actor resides in a close-knit local community that provides intensive trust and support, but might limit novel ideas and opportunities. As the ISS director dataset in Compustat only includes S&P 1500 companies, we also create a dummy to show whether companies are missing network data (dummy=1 if no network data is missing).

We assign a node in the top decile as a “core node”, and a node in bottom quintile as a “marginal node” (we use quintiles instead of deciles in the latter because there are many isolates), and a node at the 50th percentile as an “average node”. The relationship between risk structure shock and performance which the model predicts is shown in Fig S4B. It shows the same pattern as the right panel in Fig S4A, i.e., marginal nodes in the network tend to face more unfamiliar risk during the Covid-19 pandemic and higher levels of risk structure change. However, this pattern is not obvious for Covid-19 related risk structure dispersion.

**S4B Fig. Covid-Related Risk Effects for Different Nodes across Time**

**(Left: risk structure shock; Right: risk structure dispersion)**

Models S3 through S6 in Table S4 show the interaction effect for network and Covid shock. There is a positive interaction between degree centrality and risk structure shock (coefficient = 0.008, with p<0.01). This means that for each unit increase in degree centrality, the negative effect of risk structure shock on ROA will be alleviated by 0.008. This might not be a huge effect, but it does reveal a buffering of shock during turmoil. For the clustering coefficient, this effect is marginally significant 0.006 (p<0.1), confirming the existence of the community support effect. Thus, we may conclude that both high status in the general field, and close-knit local communities, provide some protection.

**S4 Table. Buffering Effect of Interlocking Directorate Network**

|  | Model S3 | Model S4 | Model S5 | Model S6 |
| --- | --- | --- | --- | --- |
|  | ROA | ROA | ROA | ROA |
| Covid-related risk structure shock | -0.111*** | -0.120*** | -0.109*** | -0.112*** |
| Covid-related risk structure dispersion | -0.001 | -0.002 | -0.001 | -0.001 |
| Covid text length | -0.001*** | -0.001*** | -0.001*** | -0.001*** |
| Proportion of covid related text | 0.012 | 0.011 | 0.012 | 0.012 |
| R&D Intensity | -1.892*** | -1.890*** | -1.903*** | -1.902*** |
| Debt to asset ratio | -0.067*** | -0.067*** | -0.070*** | -0.070*** |
| Firm Size | 0.057*** | 0.057*** | 0.052*** | 0.052*** |
| Degree centrality | -15.800*** | -15.970*** |  |  |
| Clustering coefficient |  |  | 0.01 | 0.003 |
| SP1500 dummy | 0.101*** | 0.100*** | 0.064*** | 0.065*** |
| Risk structure shock * degree centrality |  | 0.008** |  |  |
| Risk structure dispersion * degree centrality |  | -0.002 |  |  |
| Risk structure shock * Clustering coefficient |  |  |  | 0.006+ |
| Risk structure dispersion * Clustering coefficient |  |  |  | -0.001 |
| Major risk category (dummies) | Controlled | | | |
| Industry (SIC code, dummies) | Controlled | | | |
| Location: state (dummies) | Controlled | | | |
| _cons | -1.034*** | -1.028*** | -0.917** | -0.915** |
| *N* | 7957 | 7957 | 7957 | 7957 |
| R2 (overall) | 0.422 | 0.423 | 0.418 | 0.419 |

*Note:* ^+^ *p* < 0.1, ^*^ *p* < 0.05, ^**^ *p* < 0.01, ^***^ *p* < 0.001.

However, neither network measures have significant interactions with risk structure dispersion. When Covid-19 spikes the risk structure dispersion level and influences one specific business aspect, the network offers little help.

## **S5 Appendix: Technical Details of Measurement Construction and Model Building**

S5.1 Topic Modeling

Our work adopts the methods described in Blei et al. (2003) [33]. The purpose of topic modeling is to uncover the semantic themes in a collection of documents. Topic modeling assumes the existence of “topics”, a “hidden structure” situated between individual words and documents. From a statistical perspective, documents are distributions of topics, and topics are distributions of words. With data on the co-occurrence of words in documents, topic modeling aims to infer this latent structure.

To achieve this purpose, we use LDA (latent Dirichlet allocation) model. LDA assumes the following generative process for each document w in a corpus D [33]:

1. Choose N ~ Poisson (ξ);
2. Choose θ ~ Dir (α);
3. For each of the N words *Wn*:
4. Choose a topic *Zn* ~ multinomial (θ);
5. Choose a word *Wn* from *P* (*Wn|Zn, β*), a multinomial probability conditioned on the topic *Zn*.

Given the parameter α and β, the joint distribution of a topic mixture θ, a set of N topics *Z*, and a set of words *W* is given by:

$P(\theta,Z,W|\alpha,\beta)= P(\theta|\alpha)\prod_{n=1}^{N} P(Zn|\theta）P(Wn|Zn, \beta）$,

And by integrating over θ and summing over *Z*, the marginal distribution of a document can be written as:

$P(W|\alpha,\beta)=\int P\left( \theta| \alpha\right)[\prod_{n=1}^{N} \sum_{Zn} P\left( Zn | \theta\right) P(Wn|Zn,\beta)]d\theta$,

While the probability of a corpus can be written as

$P(D|\alpha,\beta)=\prod\int P\left( \theta d | \alpha\right)[\prod_{n=1}^{Nd} \sum_{Z_{dn}} P\left( Z_{dn} | \theta d \right)P(W_{dn}|Z_{dn},\beta)] d\theta_{d}$

Thus, we can estimate the parameters α and β, and calculate the topic distribution for each document and words distribution for each topic. In our analysis, we use the topic model results to construct our measurements for covid-related risk shock.

Following Blei et al. (2003) [33], we use perplexity to evaluate the goodness-of-fit of the model. Perplexity is defined as:

$$Perplexity(D_{test})=exp\left\{ -\frac{\sum_{d=1}^{M} logP(Wd)}{\sum_{d=1}^{M} Nd} \right\}$$

Which measures how successfully a trained topic model predicts new data. In Blei’s original paper, the perplexity is monotonically decreasing in the likelihood of the test data, and a lower perplexity score means better model performance. Here, after splitting all the corpus into a training set (80%) and a test set (20%) through random selection, our model achieves a perplexity score of 1340.8, which shows rather good performance compared with models in Blei’s paper.

S5.2 Pre-Processing for Text Data

Before feeding our corpus into the LDA model, we clean and pre-process the data following the standard pipeline: we tokenize the text first, delete the stop words, and conduct word lemmatization.

Word tokenization refers to the process of cutting sentences into individual word tokens. This step is completed by the word tokenize command in the nltk package in Python.

Stop words refers to the process of filtering out meaningless words because they are too common in daily language. These words include “me”, “my”, “the”, “and”, etc. Here, we use the default English stop words list in the nltk package to complete this step.

Lemmatization refers to removing inflectional endings of the words and returning the base or dictionary form of vocabulary. This helps us collapse the different inflectional forms of a lemma and make sure words with the same meaning are treated equally by the model.

S5.3 Panel Data Analysis

Panel data analysis is a statistical method, widely used in social science to analyze two-dimensional (typically cross-sectional and longitudinal) panel data. The data are collected over time and over the same individuals, and then a regression is run over these two dimensions.

In our work, we apply both the random effects and fixed effects models, as introduced by Wooldridge (2019) [54].

Fixed effects model has the form:

$$Yit=\beta*Xit+\alpha i+uit, for t=1, 2, \ldots T and i=1, 2, \ldots.N.$$

Where *αi* is the fixed intercept for each subject (i), and *Uit* is the general intercept. By averaging this equation over time (“fixed effects transformation”), we get the time-demeaned fixed effect form of the equation:

$$Yit-\bar{Yi}=\beta*\left( Xit-\bar{Xi} \right)+uit-\bar{ui}, for t=1, 2, \ldots T and i=1, 2, \ldots.N.$$

Thus, fixed effect models assume that the underlying effect is the same across all individuals, and here β estimates the within effects of x on y across time.

In comparison, if the unobserved individual heterogeneity is uncorrelated with *Xit*, the random-effects model has the form:

$$Yit=\beta0+\beta*Xit+\alpha i+uit, for t=1, 2, \ldots T and i=1, 2, \ldots.N.$$

Because of the additional *β0*, now the transformation leads to the form:

$$Yit-\theta\bar{Yi}=\beta*\left( Xit-\theta\bar{Xi} \right)+uit-\bar{ui}, for t=1, 2, \ldots T and i=1, 2, \ldots.N.$$

The random effect model allows for constant explanatory variables over time and takes between-group variation into consideration.
